# 3D printing of unsupported multi-scale and large-span ceramic via near-infrared assisted direct ink writing

**DOI:** 10.1038/s41467-023-38082-8

**Published:** 2023-04-25

**Authors:** Yongqin Zhao, Junzhe Zhu, Wangyan He, Yu Liu, Xinxin Sang, Ren Liu

**Affiliations:** 1 Key Laboratory of Synthetic and Biological Colloids, Ministry of Education, School of Chemical and Material Engineering, Jiangnan University, 214122 Wuxi, Jiangsu China; 2 International Research Center for Photoresponsive Molecules and Materials, Jiangnan University, 214122 Wuxi, Jiangsu China; 3 School of Mechanical Engineering, Jiangnan University, Wuxi, Jiangsu 214122 China; 4 Jiangsu Key Lab of Advanced Food Manufacturing Equipment and Technology, Jiangnan University, Wuxi, 213122 China

**Keywords:** Materials chemistry, Ceramics, Design, synthesis and processing, Mechanical engineering

## Abstract

In the three-dimensional printing process of ceramic with low-angle structures, additional supporting structures are usually employed to avoid collapse of overhanging parts. However, the extra supporting structures not only affect printing efficiency, but the problems caused by their removal are also a matter of concern. Herein, we present a ceramic printing method, which can realize printing of unsupported multi-scale and large-span ceramics through the combination of direct ink writing and near-infrared induced up-conversion particles-assisted photopolymerization. This printing technology enables in-situ curing of multi-scale filaments with diameters ranging from 410 µm to 3.50 mm, and ceramic structures of torsion spring, three-dimensional bending and cantilever beam were successfully constructed through unsupported printing. This method will bring more innovation to the unsupported 3D manufacturing of complex shape ceramics.

## Abstract (web summary)

In 3D ceramic printing, the need for additional supports can increase processing time and introduce defects during post-processing removal. Here, authors merge direct ink writing and up-conversion particles-assisted photopolymerization under near-infrared irradiation for support-free printing with controlled curing rates reducing material waste, printing time, and post-processing steps.

## Introduction

Ceramics have excellent mechanical properties such as structural stability, wear resistance, corrosion resistance and high temperature resistance, which are widely used in machinery, electronics, energy, aerospace, and biomedical fields^1–4^. However, constrained by the inherent brittleness and hardness characteristics of ceramic materials, precise and rapid manufacturing of complex-shaped ceramic components are difficult to achieve through traditional molding processes^5–7^. The emerge of additive manufacturing technology provides an efficient and convenient way to obtain complex ceramics with integrated structure and function, which could lead to ceramic with modern applications^8,9^.

Additive manufacturing provides a higher degree of design freedom for the manufacturing of advanced ceramics and a revolutionary impetus for the manufacturing of high-performance ceramic materials. The rapid development of additive manufacturing cannot be separated from the progress of process technology and the expansion of applicable material systems. It is currently facing a major paradigm shift towards on demand fabrication of customized functional architectures of adequate resolution with tunable chemical composition at different scales. Although well-developed processes such as stereolithography, digital light processing, or binder jetting can manufacture ceramic parts with better resolution and significantly higher production rates, there are still some challenges^10–15^. One major issue is that, due to gravity, direct manufacturing of ceramics with large-span or special-shaped structures is difficult to achieve through additive manufacturing without using additional support structures. Since the support structures need to be removed later, the so-called “what you want is what you get” advantage of 3D printing technology has not been fully concretized. Meanwhile, the additional auxiliary supports give rise to limitations in terms of poor surface quality and dimensional precision, long processing time and high cost^16–18^. Moreover, the removal of the support is prone to micro-cracks, which may lead to stress concentration when the component is loaded, increasing the risk of failure of the ceramic part. For some specially designed structures with poor openness, internal support removal is almost impossible. In order to meet the functional and lightweight requirements of ceramics, it is of great significance to explore an unsupported ceramic printing technology for large-span structures.

The UV-based in-situ photocuring-assisted direct ink writing (DIW) technology reserve the advantage of spatial and temporal control of photopolymerization. The in-suit photo-curing behavior can improve the ability of DIW to construct complex structures and enable unsupported printing of polymeric materials^19–22^. However, in the case of ceramic printing, due to the limited penetration of the UV or visible light in high solids ceramic slurries, the curing efficiency and throughput are far from satisfactory. The large amount of suspended ceramic particles in the photosensitive slurry introduces additional light scattering, refractive index and extinction coefficient^23–27^. After multiple absorption, scattering and refraction, the gradient of light energy in photosensitive medium decays, which hinders the photopolymerization process. Compared with ultraviolet light, near-infrared (NIR) light has stronger penetrating ability due to its small linear absorption and Rayleigh scattering in various media. The approach of up-conversion particles (UCPs)—assisted photopolymerization (UCAP) induced by NIR light has been successfully applied in deep photocuring, living/controlled photopolymerization, biomedical materials and 3D printing^28–35^.

Herein, we develop an approach for ceramic 3D printing, in which DIW and UCAP process is coupled together. Ceramic structures with flexible geometries and characteristic dimensions can be obtained by on-demand curing using NIR irradiation at a controlled curing rate by using this method. By adjusting the irradiation intensity and printing speed, the ceramic slurry can be cured in-situ during extrusion without the use of supports. The increasing strength and self-supporting ability of the extruded filaments improves manufacturing precision. More importantly, the flexibility of 3D printing extends to the X-Y-Z space, making it easier to print low-angle or even horizontal (down to 0^o^) overhangs without sagging or tilting defects. NIR-DIW technology further enhances the flexibility and freedom of ceramic additive manufacturing, allowing the printing of multi-material structures and enabling the unsupported printing of complex ceramic structures with high aspect ratios and large spans.

## Results

### Rheological behavior of the printing slurry

**Fig. 1:**
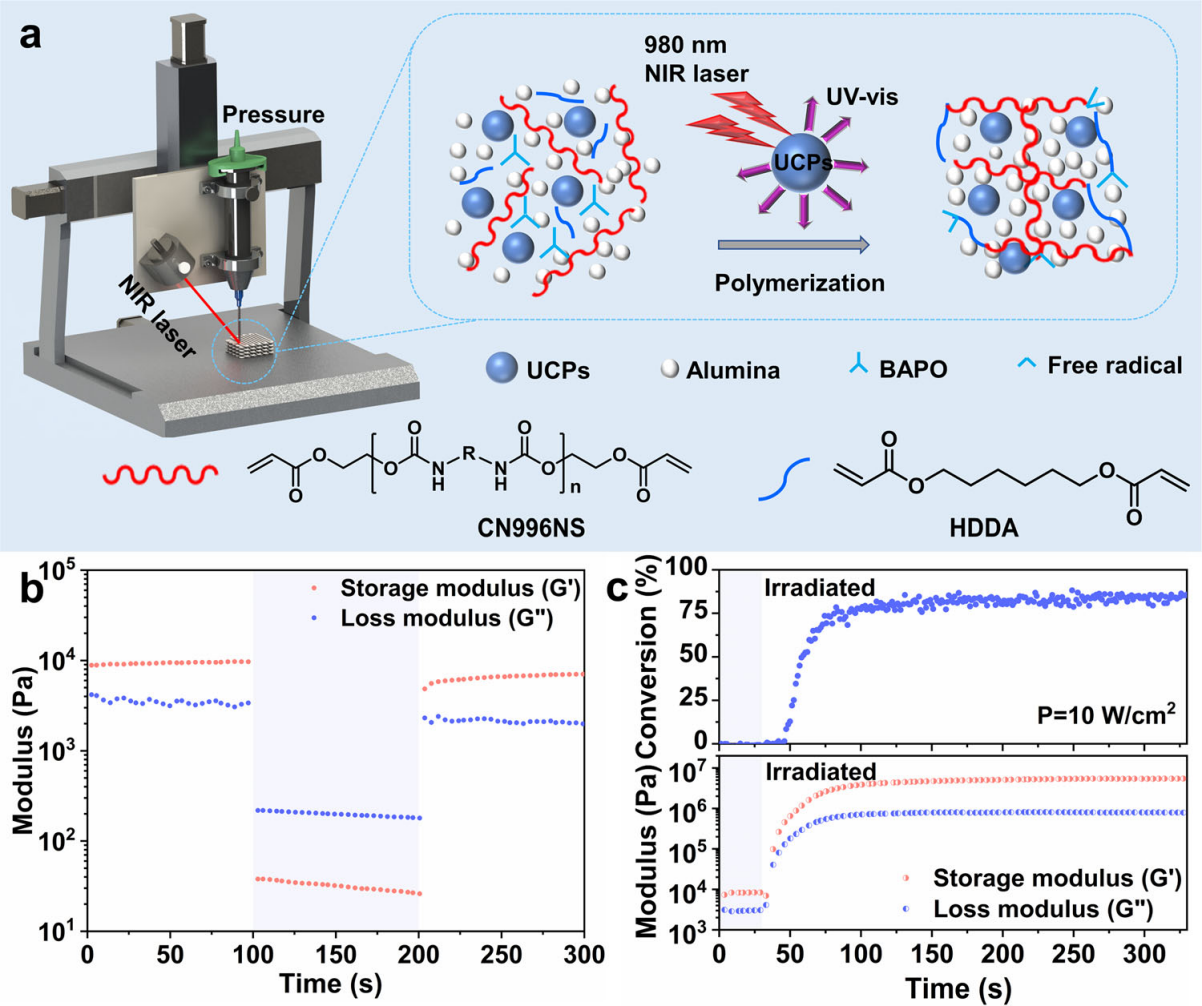
Performance of photosensitive ceramic slurry. **a** Schematic representation of NIR-assisted direct ink writing. **b** Three intervals thixotropic test showing the elastic recovery of the alumina slurry at a frequency of 1 Hz. **c** NIR-induced photopolymerization of slurry was monitored by real-time FTIR rheological analysis. The upper figure showed the double bond conversion as a function of irradiation time. The figure below showed the modulus of the slurry as a functional relationship with the irradiation time.

The schematic of NIR-assisted DIW was shown in Fig. 1a. Excellent printing structure depends on the proper rheological properties of the ink^36–38^. The maximum solid content of the printing slurry is highly dependent on the maximum extrusion pressure of the printer, the powder particle size, and the powder sphericity. The formulated slurries with 72.50 wt%, 75.00 wt% and 77.50 wt% solids all exhibited shear thinning characteristics (Supplementary Fig. 1a). However, the increase of interactions and collisions between particles in high solid content slurry leads to the increase of slurry viscosity, which will sacrifice printing speed and reduce molding efficiency. Therefore, a 75.00 wt% slurry was chosen for example validation. An excellent slurry should possess excellent thixotropic behavior, that is, the viscosity and modulus drop rapidly during high shear and recover quickly when shearing stops. Three intervals thixotropic test was used to evaluate the transition kinetics of 75.00 wt% alumina slurry from fluid flow (second interval) to elastic recovery (third interval). From the shear recovery behaviors shown in Fig. 1b, it can be seen that the average storage modulus of the slurry dropped sharply with changing from lower shear stress (30 Pa) to higher shear stress (1000 Pa). Then, under the 30 Pa shear stress of the third stage, the storage modulus quickly recovered to 62.13% of the first stage within 10 s. Three intervals thixotropic test proved that the viscosity (G” > G’) transition to elasticity (G’ > G”) was quasi-instantaneous after the release of high shear stress, which was beneficial to maintain the extruded filaments shape^39,40^. From the variation trend of the modulus of the slurry with shear stress in Supplementary Fig. 1b, it can be seen that the storage modulus of the slurry without shearing was greater than the loss modulus. The results showed that the slurry had a certain shape retention ability without collapse immediately after extrusion, and thus it had high-fidelity after in-situ curing.

### Photopolymerization kinetics

Besides the rheological behavior, the curing behavior of the slurry should be strictly controlled to provide higher shape retention for printing multi-scale and large-span structures. The extruded filaments should be cured quickly and exhibit necessary modulus to prevent collapse of the subsequent printing structure^19^. Generally speaking, the drying behavior of traditional DIW is difficult to control due to its reliance on solvent volatilization^22^. In this study, NIR-assisted curing was used to print multi-scale and large-span structures without support. The up-conversion materials can be excited with a specific wavelength of NIR light to emit ultraviolet-visible fluorescence, which was used as internal lamps for short-distance irradiation to achieve rapidly and deeply curing^33,41–43^. Figure 1c showed that the modulus and curing degree of the slurry ranged with the NIR radiation time. It can be seen that the modulus of the slurry first increased rapidly with the increase of the irradiation time, and then it was in a higher state (Fig. 1c, bottom). This result indicated that the slurry could have large curing speed and cured modulus to maintain the filaments in a preset position under NIR light.

In order to further evaluate the degree of solidification of the slurry, the FT-IR was carried out simultaneously, and the double-bond conversion was calculated according to the C = C peak evolution^44^. The double bond conversion of slurry increased rapidly and then remained unchanged with the increase of the irradiation time, and that the final conversion rate was 85.40% (Fig. 1c, top). It was consistent with the results of the real-time rheological. The absorption spectrum of the photoinitiator overlapped well with the emission spectrum of UCPs and was not competitively absorbed by alumina in the range of 350–500 nm (Supplementary Fig. 2). The influence of UCPs content on the photopolymerization process of ceramic slurry was analyzed (Supplementary Fig. 3). Therefore, the slurry could be rapidly polymerized by NIR light, resulting that the extruded filaments can be cured in-situ which composed of photocurable polymer and ceramic powder.

### Characterization of the penetration depth

The key to effective photocuring of ceramic slurry is to have enough light energy to initiate photopolymerization, which can be increased by increasing the light intensity and curing time. Our group have used silica particles as research subjects to verify the high penetration of NIR light in highly filled systems^34^. In a certain layer for NIR-induced UCAP, the incident light can be dissipated by fillers or their aggregates. The dissipation light compensates the incident light to obtain higher light intensity for photopolymerization. For UCAP processes, the lateral light source from the dissipation part further induces UCPs luminescence, which in turn affects the reaction rate of UCAP and the performance of final cure slurry.

**Fig. 2:**
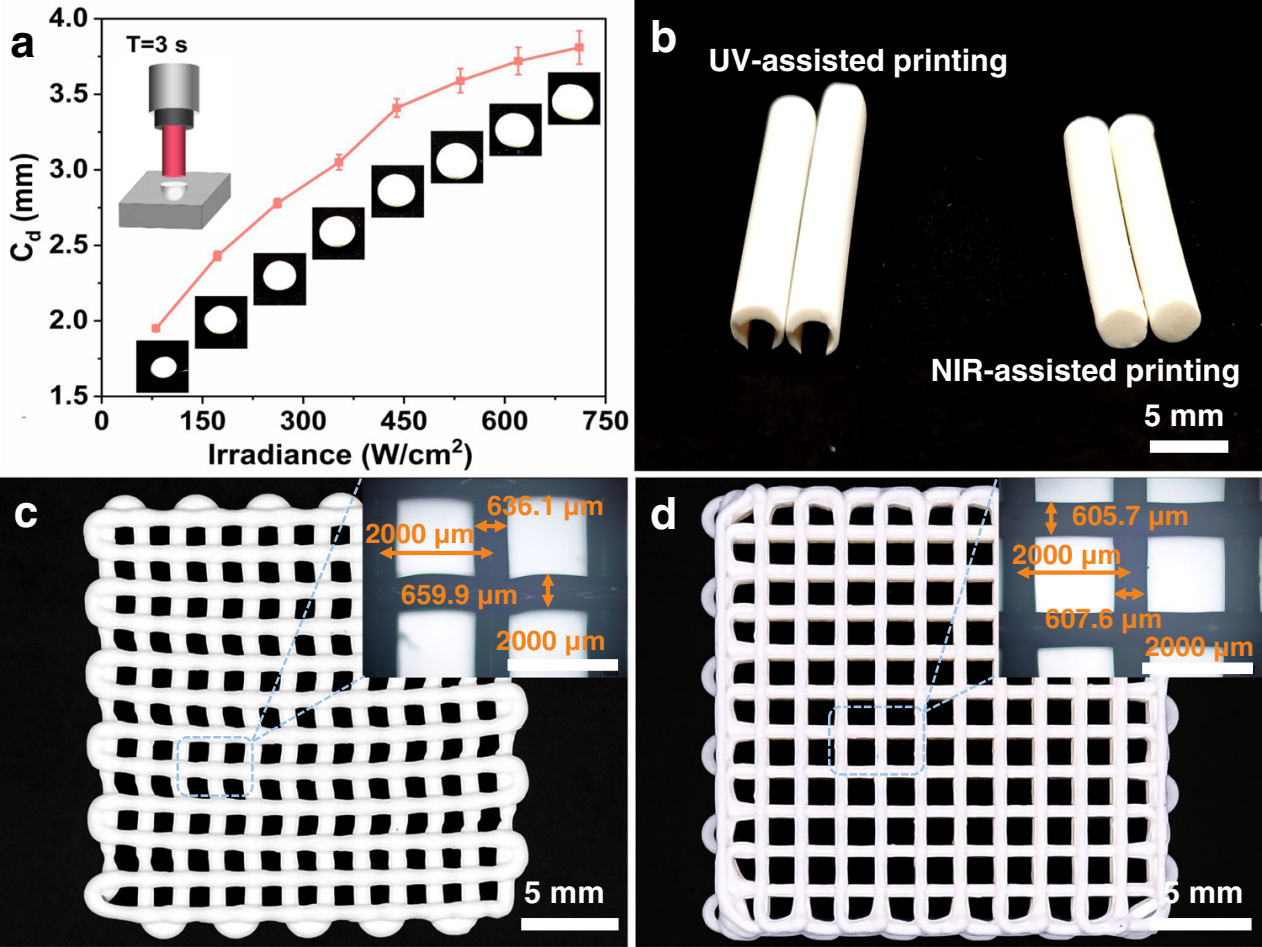
The differences between varied photopolymerization conditions. **a** The depth of the photocured slurry as a function of different NIR intensity at a constant exposure time of 3 s. The top inset is a schematic diagram of the curing depth test. The bottom inset is the actual cured objects obtained by curing for 3 s under different NIR light intensities. The curing depths (C_d_) are the heights of the actual objects. The error bars represent the standard deviations. **b** Cross-section views of large-size filaments (2.45 mm nozzle) through UV-assisted printing and NIR-assisted printing. Optical images of the structures by **c** post-cured or **d** in situ cured by NIR light. The alumina slurry was extruded through a 0.6 mm diameter nozzle at a steady speed of 5 mm/s using a gas-driven load frame.

The curing depths (C_d_) of the photosensitive ceramic slurry determined the ranges nozzles with different diameters that can be used in 3D printing. To guide the 3D printing of multi-scale structures, the effect of NIR intensities on curing depth was studied (Fig. 2a and Supplementary Fig. 4a) and compared with that of UV light (Supplementary Fig. 4b). As shown in Supplementary Fig. 4b, under the maximum power of the UV lamp (38 mW/cm^2^), the curing depth increased from 0.24 mm to 1.02 mm with the curing time ranging from 3 to 130 s. However, the increment of photocured depth would decrease with increasing curing time, since the photocured layer would hinder the penetration of irradiated light into the underlying ceramic slurry. As shown in Fig. 2a, the curing depth ranged from 1.95 mm (the NIR intensity of 82 W/cm^2^) to 3.81 mm (the NIR intensity of 713 W/cm^2^) after 3 s irradiation. It should be noted that an exposure time of 3 s could produce cured layer with a depth of about 3.81 mm, indicating that the extruded filaments could be effectively cured from the nozzle with a diameter of 3.50 mm by NIR-DIW. If necessary, larger diameter filaments would be cured using longer curing times (slower 3D printing speeds) or higher NIR light intensities. The light attenuation of the ceramic slurry was tested, and it was found that the attenuation of UV light by the slurry was significantly greater than that of NIR light (Supplementary Fig. 4c).

### Shape fidelity of printing process

To determine the effect of NIR and UV on the real-time curing of the extruded slurry, the 2.45 mm nozzle (1 mm/s) was used to print the filaments pattern on the substrate. The uncured slurry is cleaned with ethanol solvent after curing. As shown in the structure on the left side of Fig. 2b, the center and back side of the filament failed to cure due to the relatively small thickness of the slurry cured under UV light. The flat filaments affected the strength and accuracy of the printed objects, and has certain limitations for the printing of complex structures. By irradiating the printed filaments with UV-LED arrays, it is indeed possible to obtain a uniformly cured shell and to maintain the shape of the structure^22,25,27^. However, for coarser nozzles with high solid content ceramic slurry, the central part of the filament is still difficult to cure and requires further processing at a later stage. In contrast, the filaments were completely cured into a cylindrical shape when real-time curing with NIR light. Therefore, the support performance of the green body was improved, enabling high-fidelity structures to be printed without the need for support materials. The curing uniformity of NIR cured filaments was tested (Supplementary Fig. 5). The conversion is in the range of 80%-88% over the entire cross-sectional region, with a slight decrease in double bond conversion on the side away from the NIR light irradiation, but also above 80%. Combined with the results of the real-time FTIR rheological analysis of the slurry, it is clear that at this conversion rate the cured material has sufficient modulus to give a high fidelity to the printed structure. The UCPs are dispersed in the slurry without aggregates and act as an internal light source to initiate slurry curing under NIR light irradiation.

In order to make full use of in-situ photocuring-assisted 3D printing, we demonstrated how NIR light-induced photopolymerization can be integrated into DIW. The porous structure was printed by continuously depositing filaments in a stacking sequence of 0^o^/90^o^ with the NIR-induced photocuring. Both the in-situ cured and post-cured samples were printed at 5 mm/s through a 0.6 mm diameter nozzle with an applied NIR light intensity of 225.72 W/cm^2^. Post-cured samples were printed with a grid structure and then scanned at 5 mm/s using NIR light according to the print path. The printed structure undergoes shrinkage deformation during the post-curing scan of the NIR point source, triggering distortion in the adjacent uncured area (Fig. 2c). Compared with post-curing, the filaments cured by real-time (Fig. 2d) showed strong bonding and formed a very uniform square pore structure, so the size of the filaments and pores had a smaller deviation from the design. These results showed that the in-situ NIR-induced photocuring could produce good adhesion between deposited filaments and print precise ceramic structures according to the route of the printing program (Supplementary Fig. 6).

### Printing throughput of NIR-DIW

**Fig. 3:**
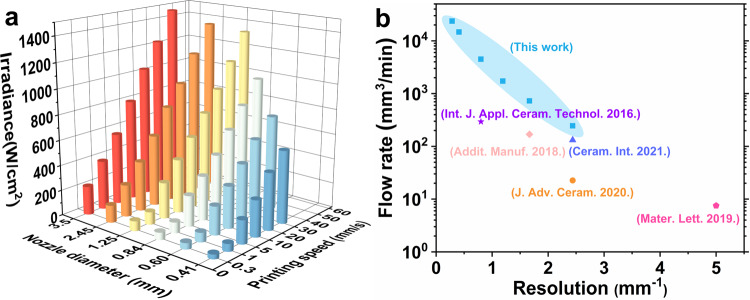
Throughput of NIR-DIW. **a** The effects of different NIR light intensities, printing speeds and nozzle diameters on curing. Nozzle diameters were plotted on the abscissa for emphasis, printing speeds were shown upward in the ordinate, with different NIR irradiance along the Z direction. Note that the required minimum light energy increases as the printing speed increases for the same size of nozzles. **b** Printing accuracy and printing throughput of mainstream DIW. The resolution is defined as 1/D, where D is the nozzle diameter. The print throughput significantly decreased during increasing the resolution.

Printing throughput represents the rapid prototyping capability of 3D printing technology. For DIW, printing throughput is defined as the actual volume of material discharged per unit time. The maximum printing speed of NIR-DIW with a fixed nozzle is highly dependent on the maximum extrusion pressure of the printer and in-situ curing. To achieve complete curing of the printed filament, the extruded material per unit time should match the in situ curing speed, which needs to be matched with a slower printing speed when using the coarse nozzle. In order to realize the rapid and high-precision printing of high solid content ceramic slurry, we studied the effects of different NIR intensities, printing speeds and nozzle diameters on the photopolymerization behaviors of extruded filaments. For any given energy dose, the maximum printing speed for a particular nozzle is shown in Fig. 3a. In order to synchronize the curing speed with the nozzle printing speed and to be able to print high fidelity structures, the extruded filaments should be properly photopolymerized by NIR light immediately after leaving the nozzle. For the same sizes of nozzles, the required minimum light energy dose increases as the printing speed increasing. For the same printing speeds, the required minimum light energy dose increases with the nozzle size increasing. It should be noted that the extruded filaments deformed and collapsed due to insufficient photopolymerization when the translation speed was greater than the curing speed. Therefore, it is reasonable to assume that the size of nozzle is rate-limiting factor for the maximum printing speed at given NIR light intensity. Based on the UCAP system, the printing throughput of NIR-DIW can be improved by changing the size and speed of the printing nozzles, while maintaining the accuracy of the printed filaments. When the nozzle sizes are 0.41, 0.60, 0.84, 1.25, 2.45, 3.50 mm, respectively, the maximum printing speed can reach 31, 43, 52, 61, 52, and 41 mm/s (Fig. 3a and Supplementary Table 1), corresponding to print throughputs of 245.44, 729.11, 1728.16, 4489.22, 14701.32, and 23655.98 mm^3^/min, respectively (Fig. 3b). Compared with other DIW printing methods^45–49^, the NIR-DIW technology uses the high penetration of NIR light and up-conversion induced photopolymerization to achieve in-situ curing of slurry, so it has a relatively large printing throughput.

### Print 3D objects without support

**Fig. 4:**
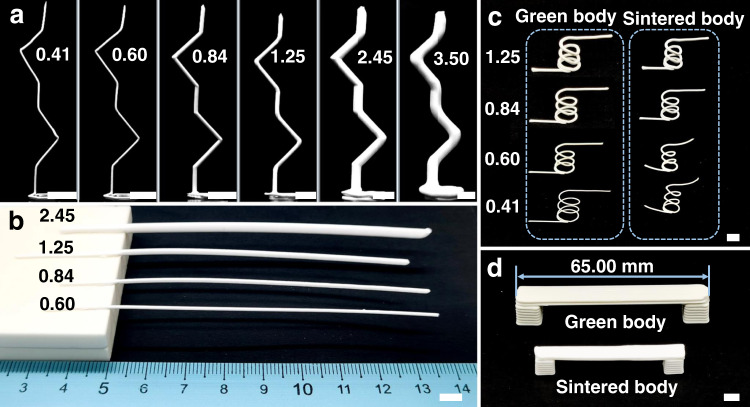
The structural stability and fidelity of the printed parts were evaluated by NIR-DIW. **a** Filaments with different size nozzles (0.41, 0.60, 0.84, 1.25, 2.45, and 3.50 mm) cured in-situ with the assistance of NIR. **b** Optical image showing the shape retention ability of the cantilever filaments (0.60, 0.84, 1.25, and 2.45 mm) printed using NIR-DIW. **c** Optical images of torsion spring structure green body and sintered body (0.41, 0.60, 0.84, and 1.25 mm) printed by NIR-DIW. **d** Green body and sintered body of flat-bridge structures are printed through NIR-DIW with a 1.25 mm nozzle. The scale bar is equivalent to 5 mm.

On the basis of examination and preliminary testing, the structural stability and fidelity of the printed parts were evaluated to verify the in-situ curing ability of the ceramic slurry. As shown in Fig. 4a, using nozzles of different diameters (0.41, 0.60, 0.84, 1.25, 2.45, 3.50 mm) to print three-dimensional curved structures at a speed of 1 mm/s, the product could be cured rapidly by adjusting different light intensity through NIR-DIW to ensure structural stability. During the slurry printing, the extruded filaments were irradiated by NIR light simultaneously to achieve in-situ curing. Higher extrusion pressure and relatively low light intensity were needed to perform real-time in-situ curing for finer nozzles. During the curing of the ceramic slurry, the surface layer of printed filament was easy to cure, while the axial part of the printed filament was weakly cured due to the attenuation of light. Therefore, for thicker nozzles printing, higher light intensity was required to achieve in-situ curing of the filaments. Figure 4a and Supplementary Movie 1-3 showed the programmed movement of the filaments along the X and Z axis. To print curved structures with tilt angles *θ*_s_ of 45°, the vertical pillars were printed first, and then translated along the X and Z axes. The filaments had excellent shape retention ability with NIR irradiation, and prevented the structure from collapsing during subsequent processing. The cured filaments with a diameter of 3.50 mm were printed by NIR assisted, indicating the versatility of NIR-DIW in multi-scale printing. We showed the bending structures with different inclination angles in Supplementary Fig. 7. These unique structures can rarely be printed without support via other additive manufacturing methods. The experimental shapes agree well with the model.

To demonstrate the possibility of printing low-angle structures with NIR-DIW, we printed cantilever filaments with 0.60, 0.84, 1.25, 2.45 mm nozzles. As shown in Fig. 4b and Supplementary Movie 4, our method can print horizontal cantilever structure with tilt angles *θ*_*s*_ of 0^o^ without support. This technology meets the needs of a large-span and has greater design flexibility. The printed cantilever structure filaments exceeded 85.00 mm and presented good shape retention with no observed viscoelastic sag. By applying NIR light-induced photopolymerization into DIW printing, we printed the high strength filaments and ceramic components with high conformability high shape retention without using any support materials. The selection of these specific structures was just to demonstrate a proof-of-concept, that is, to manufacture multi-scale complex structures with cantilever, not achievable with other additive manufacturing technologies without support materials.

In the field of additive manufacturing, there is a growing interest in the fabrication of freestanding objects. However, it is difficult to print suspended structures directly by DIW due to the viscoelastic and shear-thinning properties of the slurry itself cannot maintain the special-shaped structure. To maximize printing capabilities and generate more complex 3D structures, we extended the printing method to X-Y-Z dimensions. As an example, the torsion spring structure was printed at a constant speed of 5 mm/s (Fig. 4c). The filaments do not show obvious defects in the highly bent part. To create the sharp turn, the laser was irradiated close to 1 mm below the nozzle. When the laser is positioned too close, the light is conducted upstream through the slurry, causing the slurry to stop extruding due to curing. A well-defined torsion spring structure was constructed without any support material under the irradiation of NIR light. However, the extruded filaments collapsed due to lack of strength in the absence of NIR light (Supplementary Fig. 8). The torsion spring structures printed with 0.41 and 0.60 mm nozzles undergo high temperature creep deformation after sintering, while torsion spring structures printed with 0.84 and 1.25 mm nozzles retain their original shape after sintering.

In addition, several challenging freestanding objects were produced to demonstrate the practicality and versatility of this technology. As shown in Fig. 4d and Supplementary Movie 5, the flat bridge structure with a span of 65.00 mm was printed with 1.25 mm nozzle according to a preset 3D model. These results showed that the filaments could be quickly cured in-situ after being extruded from the nozzle with the NIR light-assisted polymerization, and some independent structures could be also printed without any support material (Supplementary Movie 6). Although someone had proven the printability of structures such as spirals by other methods^50,51^, we used NIR-DIW to quickly print ceramic structures with large-scale line features and multi-scale adjustment flexibility. Therefore, our method would be very useful in diverse fields with new functions. High-precision grid structures can be printed using a 0.41 mm nozzle (Supplementary Fig. 9). Composite structures can also be printed, where the thicker filaments provide structural properties and the finer filaments provide more functional applications (Supplementary Fig. 10). Adjusting the nozzle size not only enables multi-scale production of the same printed sample from sub-millimeter to millimeter scale, but also significantly increases the printing throughput.

### Multi-material printing

**Fig. 5:**
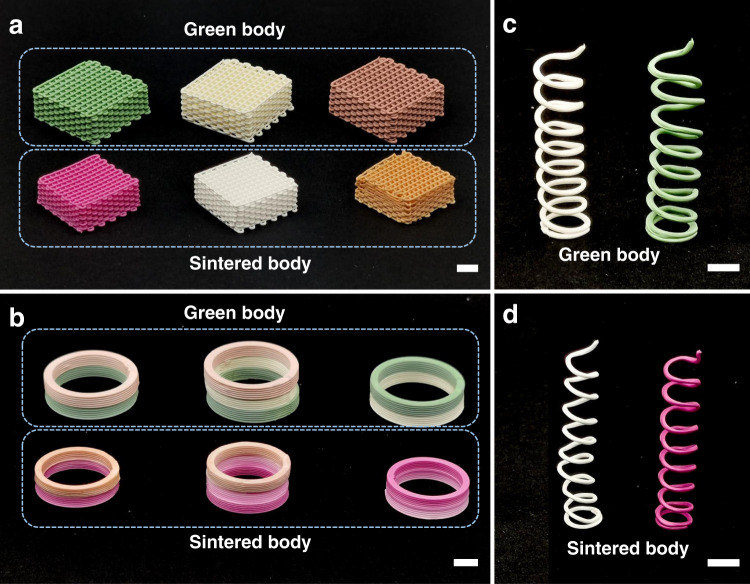
Multi-material universal printing of different slurries by NIR-DIW. **a** Photographs of grid structure, **b** discrete gradient structure, **c** spring green body, and **d** sintered spring printed with 0.60 mm nozzle. For colored ceramic slurry, the composition contained 2.00 wt% (weight of alumina) of iron red, chromium green or 3% yttrium stabilized zirconia. The scale bar corresponds to 5 mm.

Multi-material 3D printing can extend the design space to different materials and manufacture products with multiple functional properties at once without assembly. Ceramic multi-material additive manufacturing technology is still a challenging research area compared to polymer multi-material additive manufacturing technology. In addition to the different photocuring properties of ceramic materials, the anisotropic size shrinkages of ceramics and the difference in sintering temperature can easily lead to warpages, delamination, and cracks during high-temperature sintering^52^. In order to ensure a better co-sintering process for multi-material components, the sintering temperature of the different materials is matched by sintering additives, adjusting the heat treatment profile and the solid content. We showed the printing of multi-color ceramics materials (Fig. 5a, c) and the printing of discrete gradient structure (Fig. 5b) using NIR-DIW. For colored ceramic slurry, the composition contained 2.00 wt% of iron red, chromium green or 3% yttrium stabilized zirconia. The curing depth of colored ceramic slurry and the UV-vis-NIR absorption spectra of the raw materials used were tested as shown in Supplementary Fig. 11. By testing the variation of curing thickness with NIR light intensity of iron red, chromium green or 3% yttrium stabilized zirconia slurry with the addition of 2.00 wt%, it was found that the introduction of additives affects the interaction of the slurry with light and reduces the curing thickness of the slurry. The UV-vis-NIR absorption spectra of the various powders were measured, and it was found that iron red and chromium green maintained high levels of absorption in the full wavelength range, while the absorption at 980 nm for the 3YSZ and alumina used was significantly less than that in the UV band. Alumina sintered with 2.00 wt% chromium green has a red color due to the reaction of chromium oxide with alumina at high temperatures to form a solid melt. The alumina containing 2.00 wt% iron red appears brownish red after sintering. Alumina containing 2.00 wt% 3YSZ shows white after sintering (Fig. 5d). Alumina containing 2.00 wt% 3YSZ appears pink after co-sintering with alumina containing 2.00 wt% chrome green as gradient materials (Fig. 5b).

### Printing and material performance

**Fig. 6:**
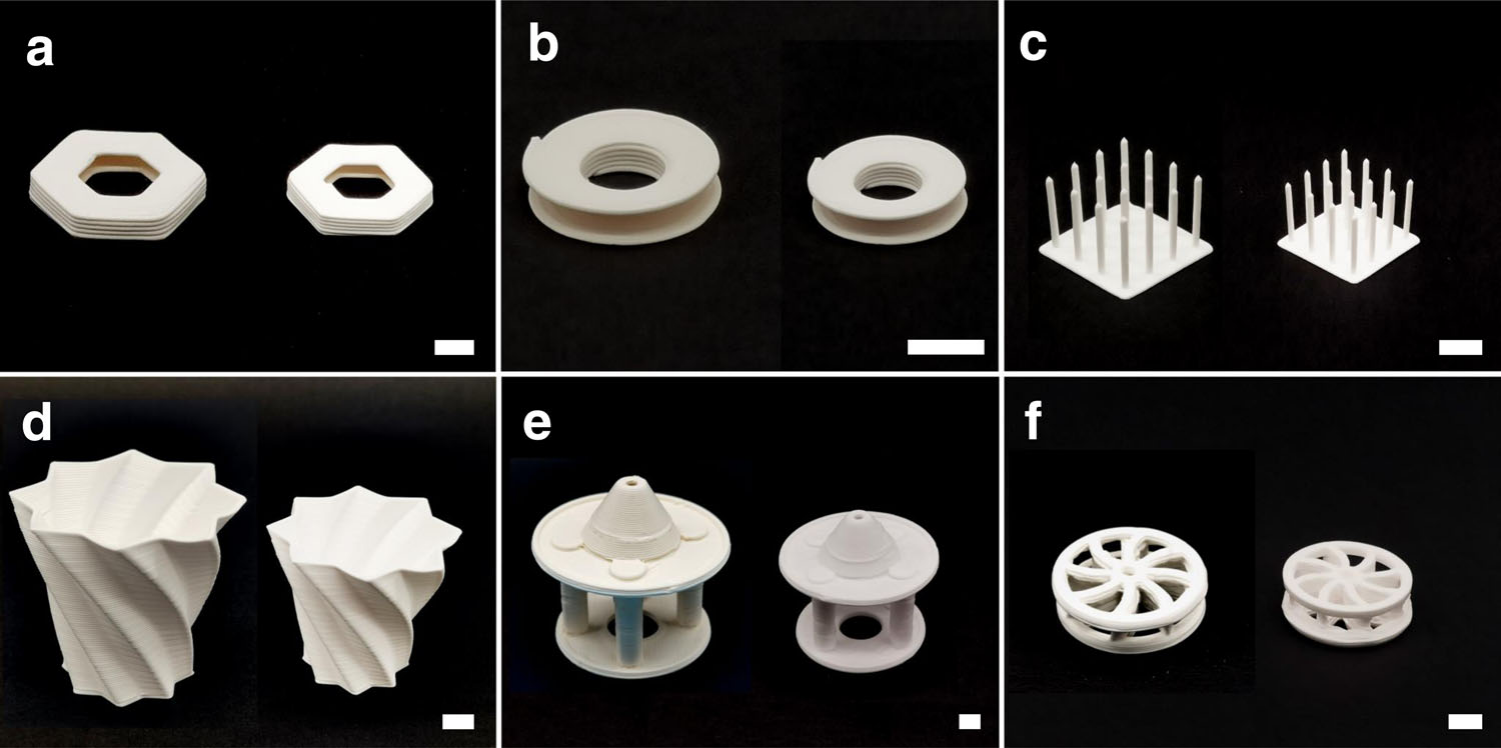
The structural stability and fidelity of the printed parts were evaluated by NIR-DIW. Different architectures of **a** overhanging hexagonal prisms, **b** pulley were printed with 0.41 mm nozzle. Different architectures of **c** freestanding pillar arrays, **d** flower-shaped container, **e** gazebo and **f** wheel were printed with 0.60 mm nozzle. The left side of each image shows the green body and the right side shows the sintered body. All scale bars are 5 mm.

In order to verify the printing capability of the freestanding objects, several special-shaped samples were printed by NIR-DIW. By applying NIR light-induced photocuring, the extruded green filaments had high strength and could be printed unsupported without collapse and deformation, as shown in Fig. 6. The printed green bodies were degreased and sintered using the procedure in Supplementary Fig. 12. The ceramic structure can shrink at a uniform rate during the sintering process, so as to maintain the self-supporting structure. The sintered ceramic samples printed with a 0.60 mm nozzle had a flexural strength of 336.45 ± 15.09 MPa, fracture toughness of 5.30 ± 0.29 MPa·m^1/2^, hardness of 17.13 ± 0.68 GPa, and a relative density of 98.32%. Scanning electron microscopy (SEM) tests were performed on sintered alumina samples of different structures (Supplementary Figs. 13–15). The sintered samples were consistent with the green body, with proper fusion of the extrusion between layers and no obvious defects were found. The samples sintered at 1600 °C for three hours showed a relatively dense microstructure with almost no pores, which led to improved ceramic properties. The micrographs of sintered parts presented growth of alumina grains compared with the original particles in the slurry, which promoted the closure of pores between grains. These results showed that NIR-DIW could produce special-shaped ceramic parts. The ceramic parts with complex shapes could be initially fabricated using optimized heat treatment processes. In addition, the sintered alumina ceramics still have specific color fluorescence under 980 nm NIR light excitation, which, we hope, would be applicated in anti-counterfeiting, photocatalysis, non-contact temperature measurement and other fields (Supplementary Fig. 16).

## Discussion

The unsupported additive manufacturing technology based on NIR-DIW opens up higher degree of freedom for ceramic additive manufacturing design. The key to this technology is not only the elimination of the support required in a typical printing process, but also brings about many other advantages such as reduced printing time, material usage and post-processing workload. At the same time, high-fidelity partial overhangs and low-angle ceramic geometries such as torsion spring, three-dimensional bending and cantilever beam have been successfully constructed. These breakthroughs further optimize the surface quality of 3D printed ceramic parts, while also eliminate the molding space occupied by support structures. With the assistance of UCAP, once the NIR radiation intensity reaches a certain value, the photosensitive ceramic slurry can be instantly solidified into a stable structure, and the printed curves can be freely stretched in space without support. The printing process is continuous and smooth, without heating and waiting for cooling. It is controllable in time and space, so that it can rapidly manufacture special-shaped structural parts. The technology allows for improved material utilization and increased design freedom. It offers advantages in terms of preparation of high aspect ratio components, rapid printing and multi-material compatibility. Although higher accuracy can be obtained by printing with smaller diameter nozzles, shape retention after sintering is significantly poorer for monofilament no-lap formed structures such as torsion springs. By optimizing the ink components and printing parameters (nozzle diameter, extrusion pressure, moving speed, light intensity, etc.), it is possible to obtain objects with higher resolution and unique appearance. It is convinced that the NIR-DIW methodology will get further expanded, and ceramic geometries produced without support will help generate more innovations and widespread the application of additive manufacturing technologies.

## Methods

### Materials

The alumina powder with an average particle diameter of 200 nm (Hefei AVIC Nano Materials Co., Ltd., Hefei, China) were used to prepare the alumina slurry in this study. UCPs (NaYF_4_:Yb, Tm) were obtained from Shanghai Ziqi Chemical Technology Co., Ltd. BYK-111 (BYK, Geretsried, Germany) was used as dispersants. Phenylbis (2,4,6-trimethyl benzoyl) phosphine oxide (BAPO) as photoinitiator was obtained from Hubei Gurun Technology (China) Co., Ltd. 1,6-Hexanediol diacrylate (HDDA) was provided by Eternal Synthetic Resins (Changshu) Co., Ltd. Difunctional polyurethane acrylate oligomer (CN996NS) was provided by Sartomer Guangzhou Chemical (China) Co., Ltd. Iron red and chrome green pigments were provided by Shanghai Kaiyin Chemical (China) Co., Ltd. 3% Yttria Stabilized Zirconia (3YSZ) was a product of Qingdao Tianyao Industrial (China) Co., Ltd. The mixed sintering aids (CaO-MgO-SiO_2_-Y_2_O_3_ mass ratio is 26.1-16.7-47.2-10) were used in the experiment. Raw materials used for sintering aids were purchased from Sinopharm Chemical Reagent Beijing Co., Ltd. The full composition of the NIR curable ceramic slurry are double functional polyurethane acrylate CN996NS accounting for 9.58%, HDDA accounting for 9.58%, 200 nm alumina accounting for 75.00%, sintering aids accounting for 2.25%, dispersant BYK111 accounting for 1.50%, UCPs accounting for 1.50%, and photoinitiator accounting for 0.59%. For colored ceramic slurry, the composition contained 2.00 wt% (weight of alumina) of iron red, chromium green or 3% yttrium stabilized zirconia.

### Procurable ceramic slurry preparation

The NIR photosensitive ceramic slurry was prepared by mechanically mixing method. The photoinitiator and the UCPs were added to the CN996NS/HDDA mixed solution, and then were dispersed with ultrasonic for 3 min to obtain photosensitive premix. The predetermined amount of alumina powder (solid loading (ϕ) = 75.00 wt%) were added into the photosensitive premix with the assistance of dispersant. The alumina slurries were vigorously mixed for 4 min using high-speed mixing machine (SpeedMixer^TM^ DAC 330-100 SE, FlackTek Limited, USA) at a speed of 2700 r/min. All the slurries were defoamed using centrifugation prior to printing.

### 3D printing methods

The home-built DIW 3D printer used for the printing experiments compiled the print paths into parametric G-code scripts. The slurry was controlled extruded onto glass or ceramic substrate and cured in real-time assisted by NIR light.

### Characterization

The degree of double-bond conversion was continuously tested by real-time ATR-FTIR (Nicolet iS10 series, Thermo Fisher). Real-time FTIR rheology was performed using a combined ATR-FTIR (Nicolet iS10 series, Thermo Fisher) and rheometer setup (HAAKE MARS60 equipped with Rheonaut annex, Thermo Fisher) with temperature controlled by a Peltier integrated unit. The NIR light source was a 980 nm NIR laser generator (FC-W-980H-50W, Changchun New Industries Optoelectronics Technology Co., Ltd). The intensity of the laser was measured by a fiber optic spectrometer (LP100, Changchun New Industries Optoelectronics Technology Co., Ltd). The modulus and three intervals thixotropic test of the ceramic slurry were measured with a rheometer (HAAKE MARS60 equipped with Rheonaut annex, Thermal Fisher). Three intervals thixotropic test was performed at a controlled temperature of 25 ^o^C and a volume of 0.04 mL to determine the time-dependent structural recovery of the slurry after shearing. The shear recovery characteristics of the slurry were evaluated according to the ratio of the modulus of the system 10 s before the third stage to the average modulus of the first stage. Microscope images of the printed green body were observed using a superfield 3D microscope (VHX-1000C, Keyence, Hong Kong). The microstructures of the sintered ceramics were characterized by scanning electron microscopy (SEM, S-4800, Hitachi, Japan, Voltage 3 kV).

The optical fiber output was to be placed at a distance of 15 cm from the samples with an incidence angle of 60^o^. The double-bond conversion (DC) was calculated as followed equation:

1 $${{{{{\rm{DC}}}}}}\left(\%\right)=\left(1-\frac{{A}_{X,t}}{{A}_{X,0}}\cdot \frac{{A}_{{ST},0}}{{A}_{{ST},t}}\right)\times 100$$

where *A*_*X,t*_ and *A*_*X,0*_ are the integrated peak areas of the functional groups in different states or initial states, and *A*_*ST,t*_ and *A*_*ST,0*_ represent the integrated reference peak areas of the non-reactive groups in different states or initial states to exclude the interference of physical factors.

A silicone gasket (4 mm thickness) with a center opening of 15 mm was positioned on the testing plate and filled with ceramic slurry. Different light intensities are used to cure the ceramic slurry. Finally, the cured structure was washed with ethanol to eliminate any residual slurry on the surface, and its thickness was measured.

## Supplementary information

Supplementary Information

Description of Additional Supplementary Files

Supplementary Movie 1

Supplementary Movie 2

Supplementary Movie 3

Supplementary Movie 4

Supplementary Movie 5

Supplementary Movie 6

## Data Availability

All data required to evaluate the conclusions of the dissertation appears in the paper and/or the Supplementary Materials. Other relevant data supporting the findings of this study are available from the corresponding author upon reasonable request. Source data are provided with this paper.

## Source data

Source Data
