# Supplementary material for: 3D printing of unsupported multi-scale and large-span ceramic via near-infrared assisted direct ink writing
Source: Nat Commun. 2023 Apr 25;14:2381. doi: 10.1038/s41467-023-38082-8 (PMC10130026; doi:10.1038/s41467-023-38082-8)
Supplement: Supplementary file 1 — Supplementary Information [file 41467_2023_38082_MOESM1_ESM.pdf]

Supplementary Information for

3D Printing of Unsupported Multi-Scale and Large-Span Ceramic via Near-Infrared Assisted Direct Ink Writing

Yongqin Zhao[1, 2], Junzhe Zhu[1, 2], Wangyan He[1, 2], Yu Liu[3, 4], Xinxin Sang[1, 2], Ren Liu[1, 2]*

1 Key Laboratory of Synthetic and Biological Colloids, Ministry of Education, School of Chemical and Material Engineering, Jiangnan University, 214122, Wuxi, Jiangsu, China.

2 International Research Center for Photoresponsive Molecules and Materials, Jiangnan University, 214122, Wuxi, Jiangsu, China.

3 School of Mechanical Engineering, Jiangnan University, Wuxi, Jiangsu, 214122, China.

4 Jiangsu Key Lab of Advanced Food Manufacturing Equipment and Technology, Jiangnan University, Wuxi, 213122, China.

*Corresponding author. Email: liuren@jiangnan.edu.cn

This PDF file includes:

Supplementary Figures 1 to 16

Supplementary Table 1

Supplementary Figures

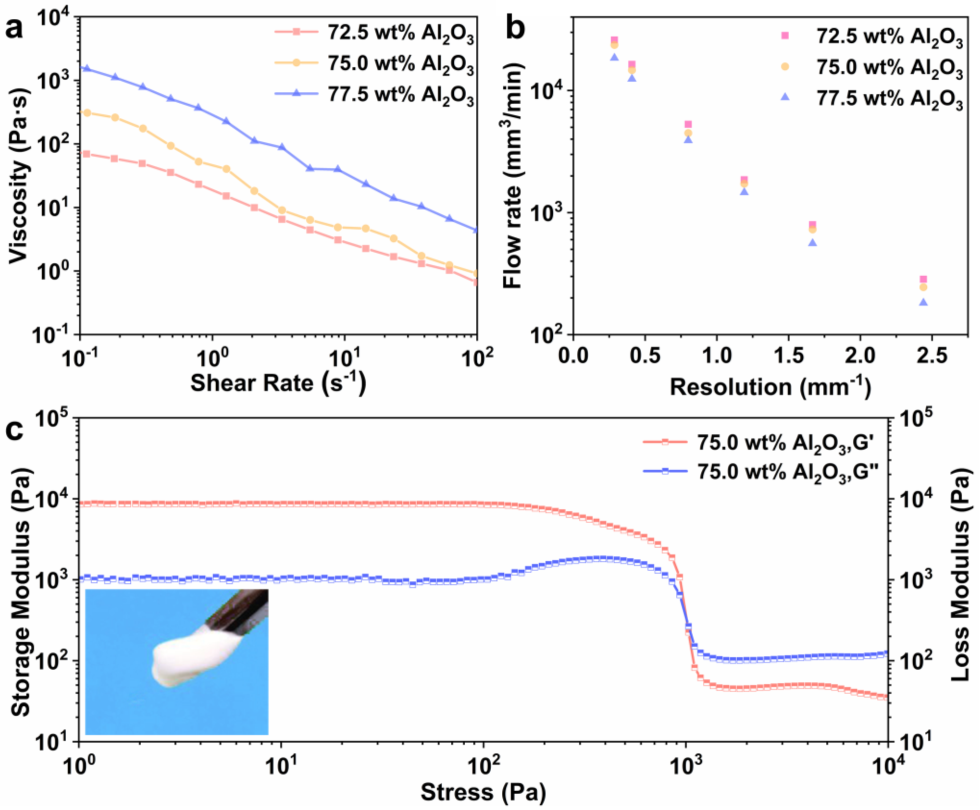

Supplementary Figure 1. Rheological properties and print throughput of alumina slurries. a Complex viscosity of alumina slurries with different solids content at different oscillation frequencies. In this manuscript, the alumina powder of 200 nm is used, and the maximum solid content can be 77.50 wt%. **b** Comparison of print throughput for different solids content slurry. However, the increase of interactions and collisions between particles in high solid content slurry leads to the increase of slurry viscosity, which will sacrifice printing speed and reduce molding efficiency. Therefore, a 75.00 wt% slurry was chosen for example validation in the paper. **c** Shear modulus G' and G" of the printing slurry under different shear stress. Variation of slurry modulus at a shear frequency of 10 Hz and a shear stress of 1-10^4 Pa. The modulus first shows a stable plateau. With the increase of stress amplitude, the two curves of G′ and G" will rapidly decrease and produce intersection points.

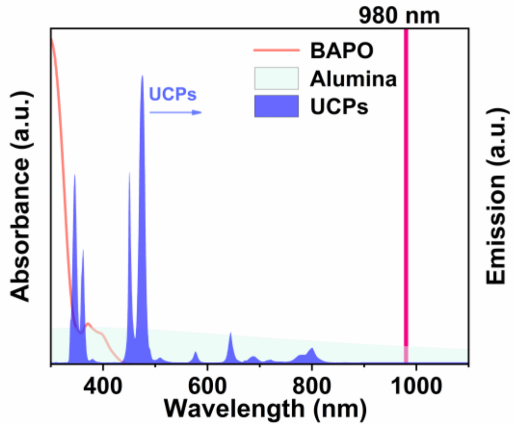

Supplementary Figure 2. Spectral matching. Overlap of the absorption spectra of the photoinitiator BAPO and 0.1% 200 nm alumina in dimethylformamide and the emission spectrum of UCPs in hexane. The fluorescence emission spectrum of UCPs was tested with the CARY Eclipse fluorescence spectrophotometer of Varian Co., Ltd. The excitation was performed using a modified 980 nm laser emitter equipped with a scanning range of 300-1100 nm in 1.0 nm steps. UV-vis-NIR absorption spectra were measured using an instrument model TU-1901 double-beam UV-Vis spectrophotometer manufactured by Lambda Company of Japan. The wavelength range was 300-1100 nm, the sampling interval was 1.0, the scanning speed was fast, and the photometric values were absorption values.

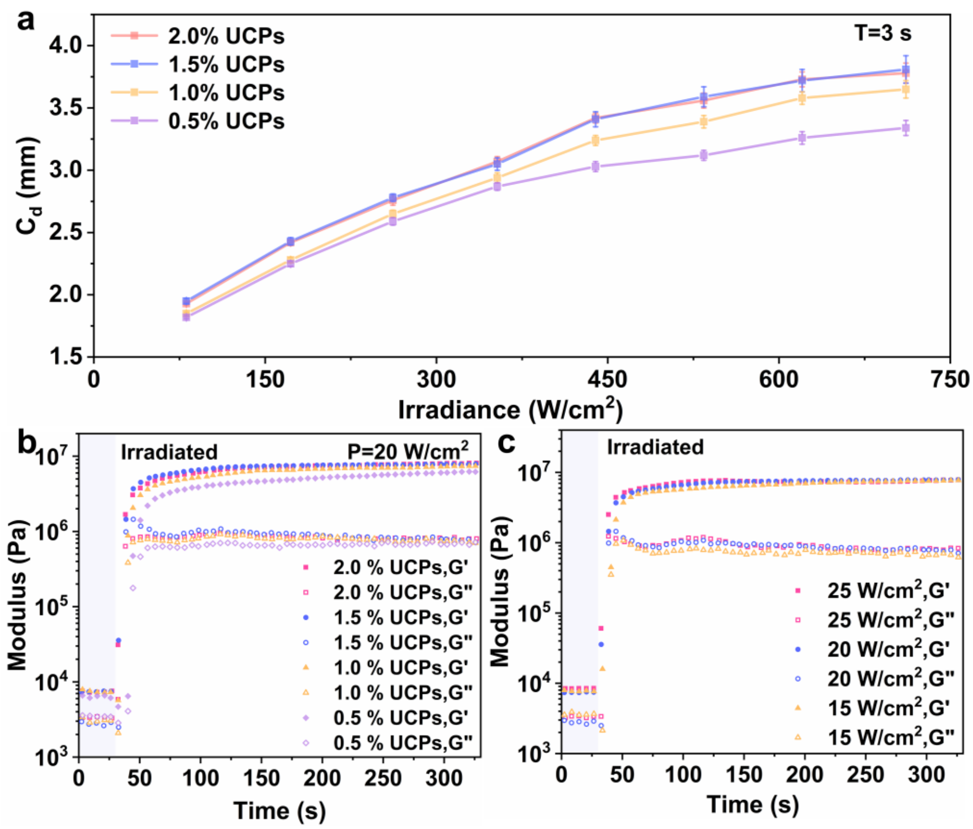

Supplementary Figure 3. Effect of UCPs content on the curing performance of slurry. a The curing depths (C_d) of photocured slurry with different UCPs content as a function of different NIR intensities at a constant exposure time of 3s. The error bars represent the standard deviations. **b** The NIR induced photopolymerization of the slurry was monitored by real-time rheological analysis under the NIR light intensity of 20 W/cm^2; **c** Real-time rheological analysis of slurry containing 1.5 wt% UCPs under different NIR light intensities.

The effect of UCPs content on the photopolymerization process was analyzed, and the curing thickness of the slurry was tested using different NIR light intensities under 3s irradiation. With the increase of irradiation power, the curing thickness first increased and then gradually entered the plateau (Supplementary Fig. 3a). The real-time rheology of the slurry was tested using a NIR light intensity of 20 W/cm^2 and irradiation started at 30 s for 300 s. The curing rate was relatively slowest for the 0.5 wt% UCPs and started to accelerate for the 1.0 wt% UCPs (Supplementary Fig. 3b). The ceramic slurry with less than 0.5 % UCPs content result in lower cure rates and prolong the plateau period of cure. 2.0 wt% and 1.5 wt% UCPs had similar curing thickness and curing rate, indicating that the UCPs were close to saturation concentration. The real-time rheology

of 1.5 wt% content UCPs slurry was tested using different NIR light intensities with irradiation starting at 30 s for 300 s. As the NIR light intensity increased, the slurry had faster initial reaction acceleration and the final storage modulus was close for the three different light intensities (Supplementary Fig. 3c).

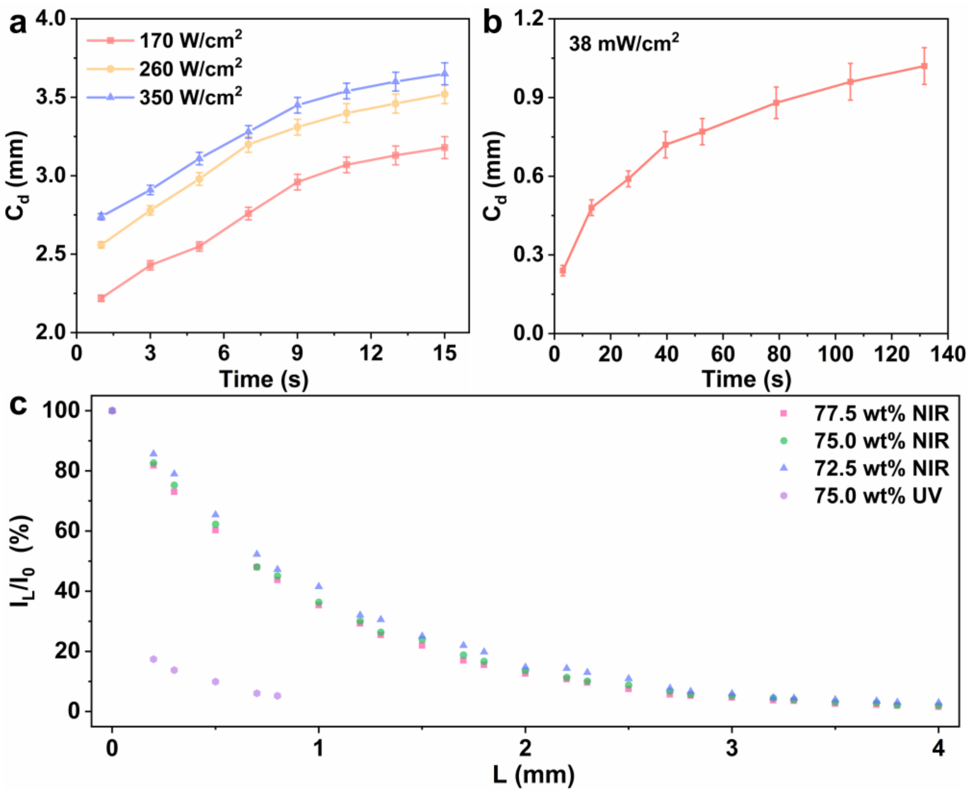

Supplementary Figure 4. Curing properties and light attenuation of ceramic slurry. a Variation of curing depth (C_d) of slurry with irradiation time at different NIR light intensities. **b** Curing performance of alumina slurry under UV light. In order to make the extruded filaments fast curing, the UV lamp was operated at maximum power. The curing depths (C_d) of the photocured slurry as a function of different exposure times at a constant UV power of 38 mW/cm^2. The error bars represent the standard deviations. **c** I_L/I_0 as a function of depth for the ceramic slurry. I_0 is the light intensity of the slurry surface and I_L is the light intensity of the ceramic slurry at L. The slurry has strong attenuation to UV light.

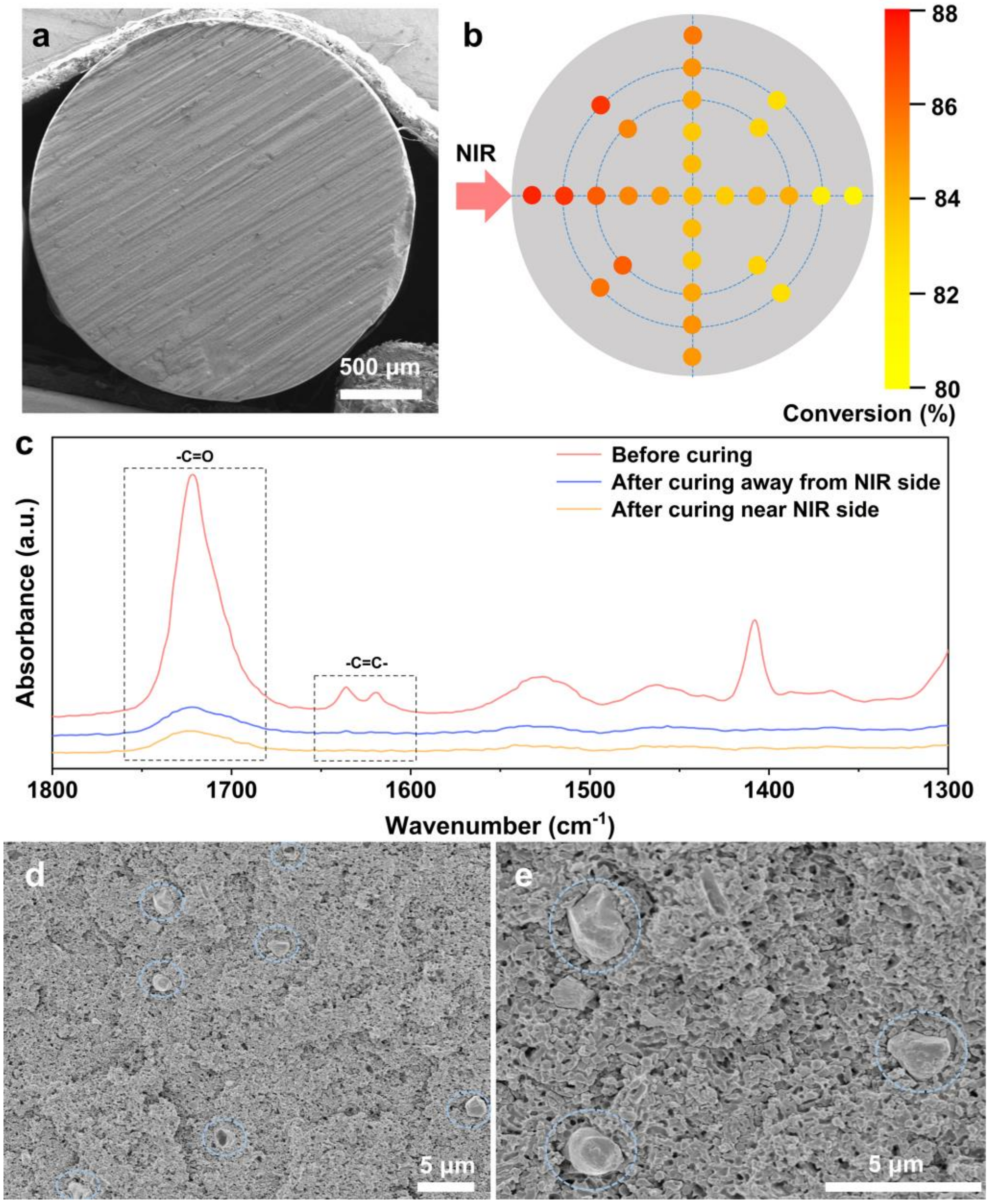

Supplementary Figure 5. Curing performance of different areas. a Cross section of a large filament (2.45 mm nozzle, speed 1 mm/s, light intensity 252.79 W/cm^2) printed by NIR-DIW. **b** Double bond conversions in different regions. An amount of material was taken at various area points of the cross section and mixed and ground with potassium bromide at a ratio of 1:100. The Fourier Transform Infrared (FT-IR) spectra were tested using the pressed slice method. According to the calculation method of the characterization part in the manuscript, the distribution diagram of double bond conversion rate at each region point was obtained. **c** FTIR spectra of points in the region near the NIR side and away from the NIR side and the FTIR spectra of the slurry before curing. SEM images with different magnification of UCPs distribution in green body are shown in **d** and **e**.

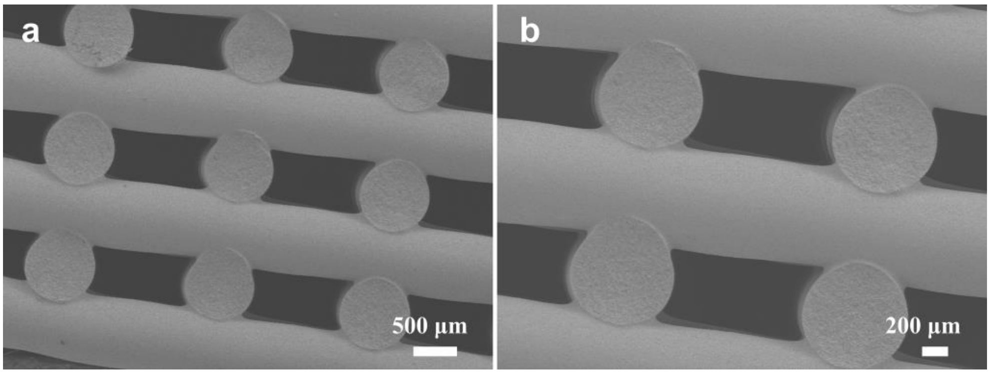

Supplementary Figure 6. Adhesion between filaments of grid green body structure. **a-b** SEM images of the printed grid green body section using a 0.84 mm nozzle.

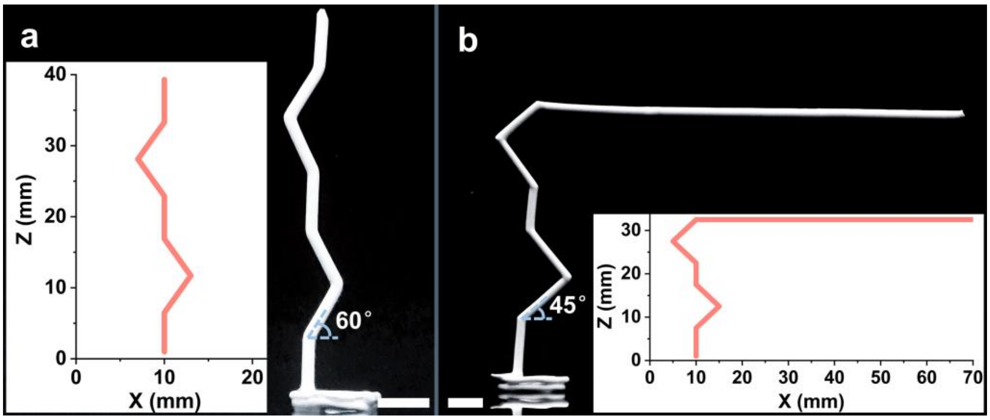

Supplementary Figure 7. Models and corresponding three-dimensional curved structures are printed with NIR-DIW through 1.25 mm nozzle. a To print curved structures with tilt angles θ_s of 60°. **b** To print curved structures with tilt angles θ_s of 45°, and then print 60.00 mm cantilever filaments along the X axis. All scale bars are 5 mm.

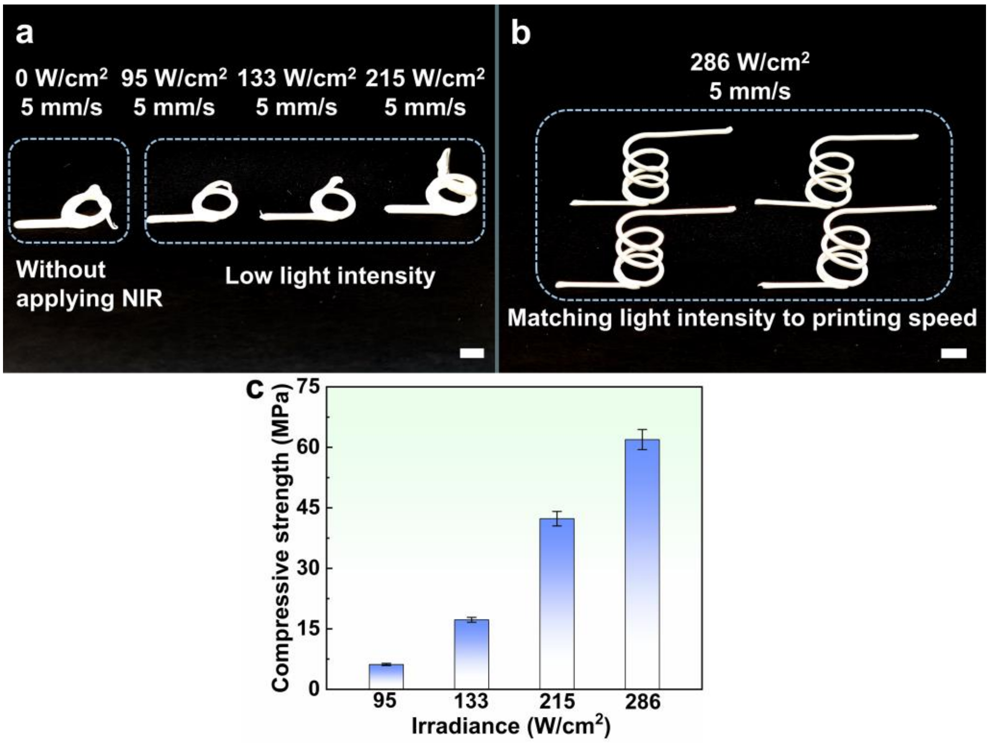

Supplementary Figure 8. Influence of NIR light intensity on conformal properties. **a-b** The effect of NIR light intensity on printing performance with 1.25 mm nozzle for torsion spring structures. For thicker nozzles printing, higher light intensity was required to achieve in-situ curing of the filaments. The scale bar is equivalent to 5 mm. **c** The compressive strength test of the printed cube grid structure with a side length of 20 mm (filling rate of 60%) under different light intensities. While printing with large diameter nozzles, the strength of the cured filament itself and fewer interfaces combine to achieve stronger printed structures. This is also another advantage of using the high penetration of NIR light and the up-conversion fluorescence induced in-situ photopolymerization. The error bars represent the standard deviations.

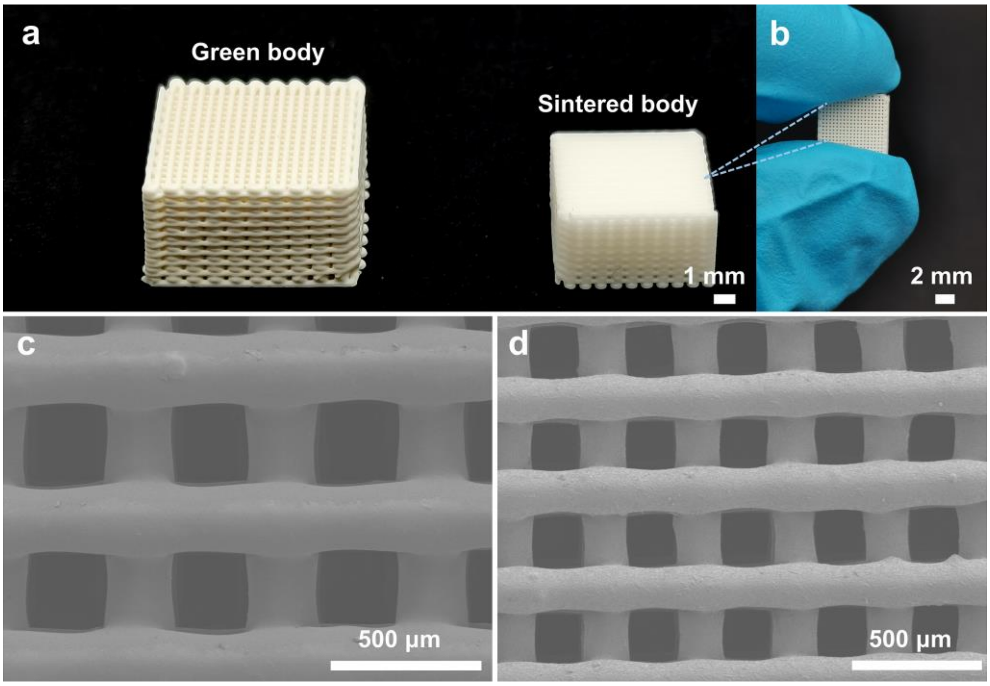

Supplementary Figure 9. Fine structure printing. a Optical images of grid green and sintered bodies printed using a 0.41 mm nozzle; **b** Elevation of the grid sintered body; **c** SEM images of the grid green body; **d** SEM images of the grid sintered body.

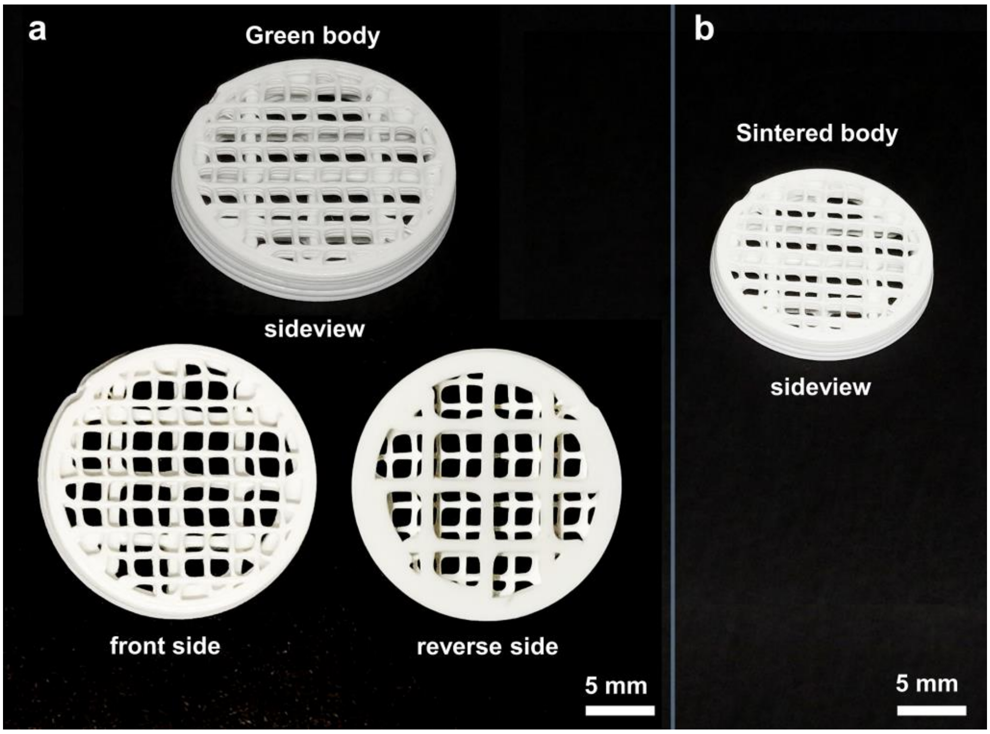

Supplementary Figure 10. Multi-scale structural printing. The 0.41mm and 0.84mm nozzles were used to print the **a** green body and **b** sintered body across the scale respectively. The scale bar corresponds to 5 mm.

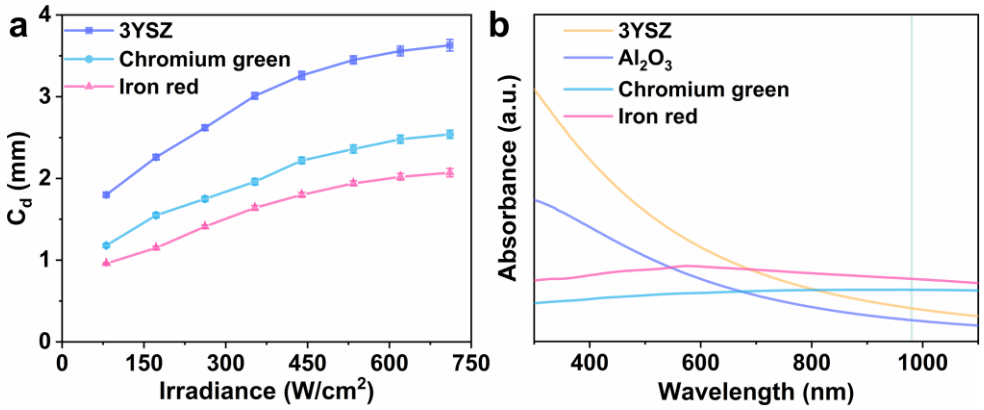

Supplementary Figure 11. Curing and light absorption properties of multi-filler slurry. a The curing depths (C_d) of the photocured slurry as a function of different NIR intensity at a constant exposure time of 3 s. The error bars represent the standard deviations. For ceramic slurry, the composition contained 2.00 wt% (weight of alumina) of iron red, chromium green or 3% yttrium stabilized zirconia. **b** The UV-vis-NIR absorption spectra of the various powders. The test conditions were 0.01% concentration of the powder dispersed in ethanol solution.

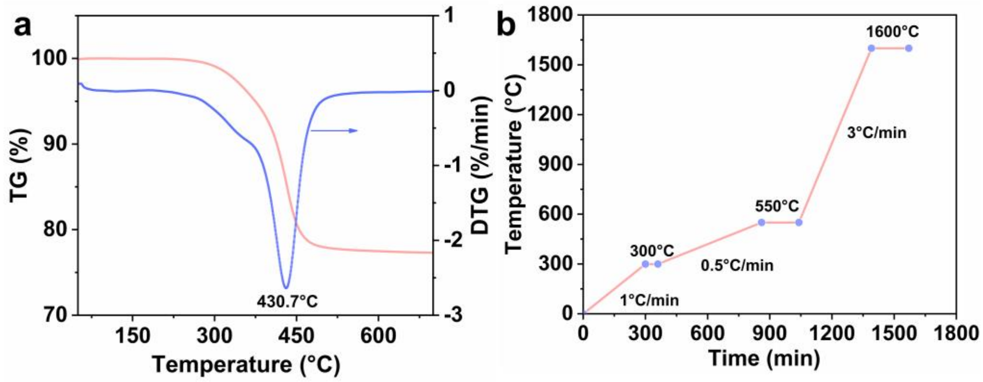

Supplementary Figure 12. Thermogravimetric analysis and sintering process of alumina green body. a TG/DTG curves, and **b** schedule for debinding and sintering of the printed green body. The weight loss of the green body was characterized by Thermogravimetric Analyzer (TGA, 1100SF, Switzerland) at the range of 50°C to 700°C. The heating rate was 15°C/min.

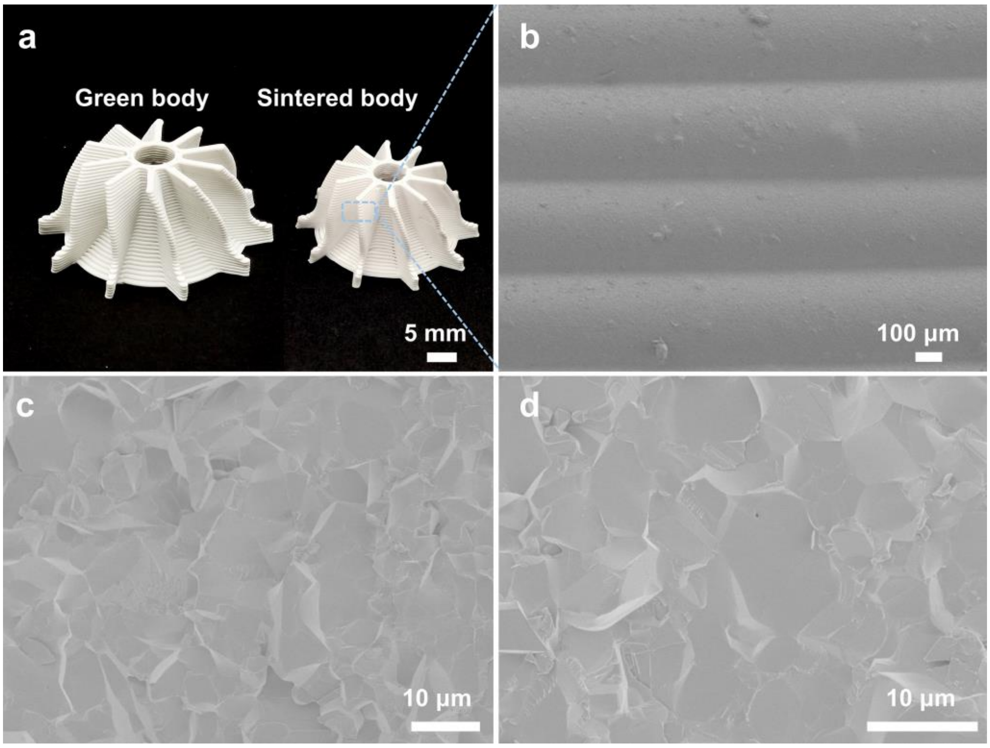

Supplementary Figure 13. SEM analysis of impeller structure. a Optical images of impeller green body and sintered body printed using a 0.60 mm nozzle. **b** SEM images show a strong bond between the straight filament structures. **c-d** SEM images of sintered ceramic sections at different magnification.

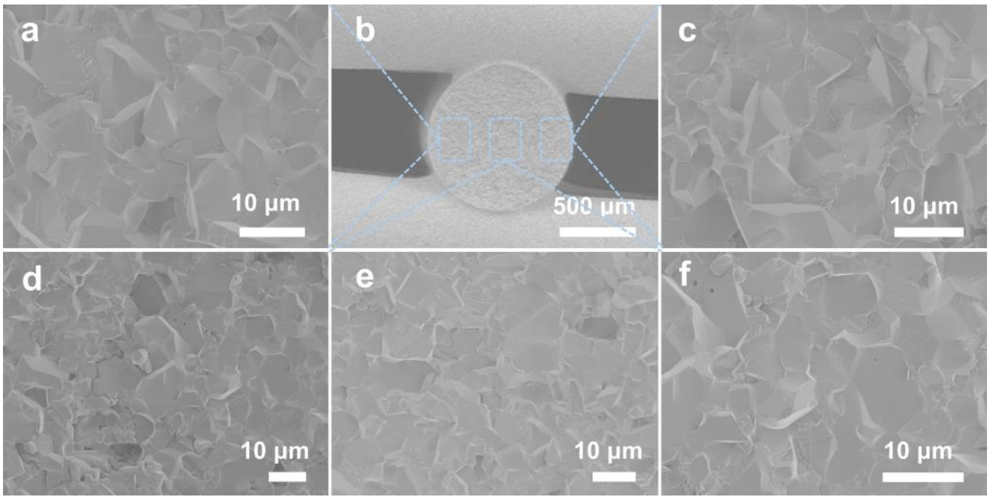

Supplementary Figure 14. SEM analysis of a sintered grid structure printed with a 1.25 mm nozzle. a-f SEM images of the cross-section of sintered ceramic structures.

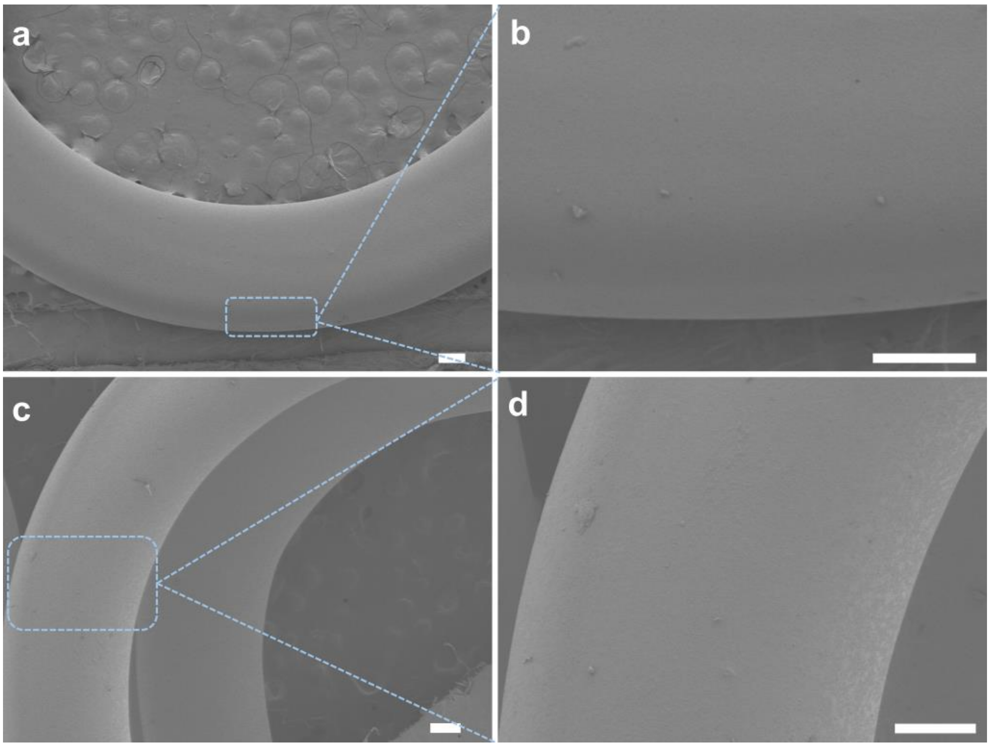

Supplementary Figure 15. SEM analysis of sintered torsion spring structures printed with a 1.25 mm nozzle. a-d SEM images of the spring turning of the sintered sample. All scale bars are 200 μm.

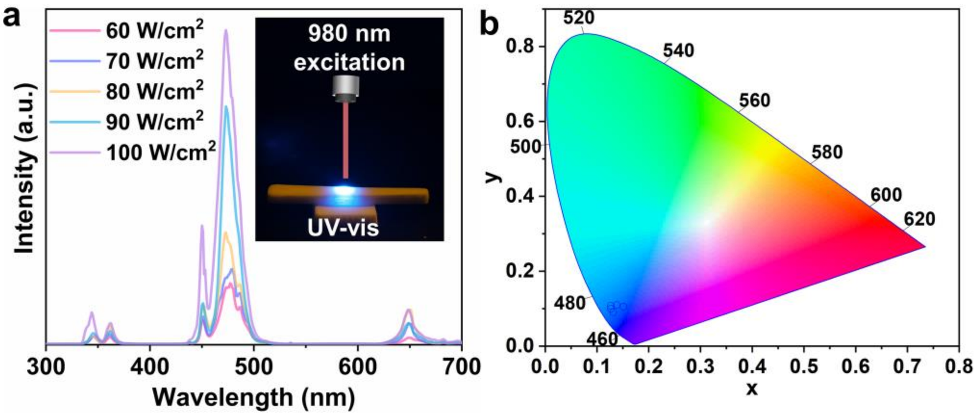

Supplementary Figure 16. Ceramic fluorescent emission. a Up-conversion emission spectra and **b** color coordinates of sintered alumina ceramics containing UCPs under 980 nm NIR laser excitation. The higher the excitation power, the stronger the up-conversion luminescence intensity. On the chromaticity coordinate of xy, a certain color is represented by (x, y). Under NIR excitation, the up-conversion fluorescence of sintered ceramics is mainly blue light, corresponding to the (x, y) values (0.1267, 0.1004) (0.1268, 0.1081) (0.1313, 0.0910) (0.1380, 0.1099) (0.1512, 0.1052) in CIE 1931.

Supplementary Table 1. The effects of different NIR light intensities, printing speeds and nozzle diameters on curing.

Nozzle diameter (mm)	Printing speed (mm/s)	Irradiance (W/cm^2)
0.41	0.3	36.28
0.41	1	59.73
0.41	5	186.03
0.41	10	288.87
0.41	20	453.06
0.41	31	577.55
0.6	0.3	48.91
0.6	1	74.17
0.6	5	225.72
0.6	10	333.98
0.6	20	463.88
0.6	30	613.64
0.6	43	748.96
0.84	0.3	57.93
0.84	1	84.99
0.84	5	243.77
0.84	10	352.02
0.84	20	478.32
0.84	30	640.70
0.84	40	803.08
0.84	52	979.90
1.25	0.3	77.78
1.25	1	99.43
1.25	5	285.26
1.25	10	427.80
1.25	20	570.34
1.25	30	730.91
1.25	40	893.30
1.25	50	1091.76
1.25	61	1308.27
2.45	0.3	135.51
2.45	1	252.79
2.45	5	397.13
2.45	10	568.53
2.45	20	767.00
2.45	30	929.38
2.45	40	1145.89
2.45	52	1362.40
3.5	0.3	225.72

3.5	1	388.11
3.5	5	568.53
3.5	10	803.08
3.5	20	1037.64
3.5	30	1236.10
3.5	41	1470.65
