# Supplementary material for: 3D printing of unsupported multi-scale and large-span ceramic via near-infrared assisted direct ink writing
Source: Nat Commun. 2023 Apr 25;14:2381. doi: 10.1038/s41467-023-38082-8 (PMC10130026; doi:10.1038/s41467-023-38082-8)
Supplement: Supplementary file 2 — Description of Additional Supplementary Files [file 41467_2023_38082_MOESM2_ESM.docx]

**File Name: Supplementary Movie 1.**

Description: Printing of three-dimensional curved structures used a 0.60 mm nozzle (played at 5x speed).

**File Name: Supplementary Movie 2.**

Description: Printing of three-dimensional curved structures used a 1.25 mm nozzle (played at 5x speed).

**File Name: Supplementary Movie 3.**

Description: Printing of three-dimensional curved structures used a 3.50 mm nozzle (played at 5x speed).

**File Name: Supplementary Movie 4.**

Description: Printing of cantilever filaments used a 2.45 mm nozzle (played at 5x speed).

**File Name: Supplementary Movie 5.**

Description: Printing of flat bridges used a 1.25 mm nozzle (played at 3x speed).

**File Name: Supplementary Movie 6.**

Description: Printing of freestanding pillar arrays used a 0.60 mm nozzle (played at 1x speed).
